# Editorial: Coma and disorders of consciousness: an overview

**DOI:** 10.3389/fnhum.2024.1383116

**Published:** 2024-02-20

**Authors:** Shraddha Mainali, Christa O'Hana Nobleza, Brian L. Edlow, Leonard Polizzotto, Neha Dangayach, Aarti Sarwal, Olivia Gosseries

**Affiliations:** ^1^Department of Neurology, Virginia Commonwealth University, Richmond, VA, United States; ^2^Department of Neurology, Baptist Memorial Healthcare, Memphis, TN, United States; ^3^Department of Neurology, Center for Neurotechnology and Neurorecovery, Massachusetts General Hospital and Harvard Medical School, Boston, MA, United States; ^4^Athinoula A. Martinos Center for Biomedical Imaging, Massachusetts General Hospital and Harvard Medical School, Charlestown, MA, United States; ^5^Department of Biomedical Engineering, Worcester Polytechnic Institute, Worcester, MA, United States; ^6^Neurocritical Care Division, Mount Sinai Health System, New York, NY, United States; ^7^Wake Forest Baptist Health Center, Winston-Salem, NC, United States; ^8^Coma Science Group, GIGA Consciousness, University of Liege, Liege, Belgium; ^9^Centre du Cerveau, University Hospital of Liege, Liege, Belgium

**Keywords:** coma, disorders of consciousness, acute brain injuries, spinal cord stimulation, transcranial magnetic stimulation, rehabilitation, diagnosis, treatment

The exploration of coma and disorders of consciousness (DoC) remains a significant frontier in neurology, characterized by its complex challenges and the critical need for innovative therapeutic interventions. This Research Topic stems from the Curing Coma Campaign and its World Coma Day. The Curing Coma Campaign is the first global public health initiative to unify the concept of coma as a treatable medical entity with the goal of promoting recovery of consciousness through early intervention and long-term support. The assemblage of 13 scholarly articles in this Research Topic provides a broad perspective on the contemporary state of research and clinical practice pertaining to this field. Collectively, these studies shed light on the complicated, dynamic, and progressive nature of DoC, providing an overview of cutting-edge research that underscores novel treatment approaches, the critical timing and methodologies of rehabilitation, recent advancements in diagnostic tools, and the multifaceted ethical considerations necessary in clinical care.

Improving the diagnosis of patients with DoC is of paramount importance given the high rate of behavioral misdiagnosis. The critical importance of accurate diagnosis and assessment in DoC is explored in “*Clinical application of recommendations for neurobehavioral assessment in disorders of consciousness: an interdisciplinary approach*.” This review by Murtaugh et al. advocates for the use of standardized neurobehavioral rating scales to improve diagnostic accuracy, thereby facilitating more effective treatment planning and management. The article highlights the challenges of diagnosing DoC and the potential for improved outcomes through more precise assessment methodologies. Next, two studies of this Research Topic used auditory paradigms and EEG to improve diagnosis and prognosis at the bedside. Ferré et al. investigate the preservation of self-recognition capabilities in DoC patients in their original work “*Self-processing in coma, unresponsive wakefulness syndrome and minimally conscious state*.” This novel research on 112 DoC patients (acute and subacute, >3 months) suggests that the ability to process self-referential auditory stimuli may serve as an early indicator of potential consciousness recovery, offering a new perspective on the assessment and prognostication of DoC patients. On their side, Binder et al. explore the evoked auditory responses to the chirp-modulated auditory stimulation as a potential biomarker for assessing awareness in prolonged DoC patients (*n* = 62) in their paper “*Diagnosing awareness in disorders of consciousness with gamma-band auditory responses*.” This pioneering approach offers a promising new method for evaluating consciousness, enhancing the diagnostic capabilities in the field of DoC research.

In addition to enhancing diagnosis and prognosis, it is crucial to improve the management and therapeutic intervention for DoC patients. The indispensable role of specialized rehabilitation is emphasized in “*Specialized intensive inpatient rehabilitation is crucial and time-sensitive for functional recovery from disorders of consciousness*.” This study by Zhang et al. on 137 DoC patients (acute, subacute and chronic stages) advocates for the timely initiation of rehabilitation interventions, highlighting the window of opportunity in which these treatments can have the most significant impact on recovery outcomes. The research underscores the need for early, active management and intensive therapies to maximize the therapeutic benefits for DoC patients. Zandalasini et al. synthesized the available research on the impact and management of neurogenic bowel dysfunction (NBD) in patients with acute brain injury (*n* = 1.507) in their scoping review titled “*Bowel dysfunctions after acquired brain injury: a scoping review*.” It reveals that oral laxatives are commonly used for treatment, yet there is a notable gap in instrumental assessment methods for incontinence. Highlighting the challenge of managing overlapping symptoms of NBD, the authors advocate for a collaborative strategy between the fields of rehabilitation and gastroenterology to enhance the diagnosis and treatment of NBD.

The utilization of neuromodulation techniques is gaining interest in both scientific and clinical communities working with DoC. In their work “*Effectiveness on level of consciousness of non-invasive neuromodulation therapy in patients with disorders of consciousness: a systematic review and meta-analysis*,” Liu et al. offers a comprehensive meta-analytical review of the efficacy of non-invasive neuromodulation therapies, such as transcranial Direct Current Stimulation (tDCS) and Transcranial Magnetic Stimulation (TMS), for prolonged DoC patients. By synthesizing data from multiple studies on a total of 345 patients, this review identifies key factors that influence treatment effectiveness, providing guidance for future research and clinical practice in developing more targeted and personalized therapeutic interventions.

The efficacy of repetitive transcranial magnetic stimulation (rTMS) is meticulously examined by Xu et al. in “*Repetitive transcranial magnetic stimulation over the posterior parietal cortex improves functional recovery in nonresponsive patients: A crossover, randomized, double-blind, sham-controlled study*.” This investigation shows that 10 Hz rTMS targeting the posterior parietal cortex significantly enhanced functional recovery in 20 unresponsive patients (>28 days and <1-year post-insult), suggesting a promising non-invasive approach to treating DoC. Although a small-scale study, the thoughtful study design supports the need for further exploration of rTMS as a therapeutic tool in the neurorehabilitation of DoC patients.

Three studies within this Research Topic investigate spinal cord stimulation (SCS) as a potential therapy for DoC. The manuscript “*Short-term spinal cord stimulation in treating disorders of consciousness monitored by resting-state fMRI and qEEG: The first case report*” by Yang et al. introduces a pioneering case where SCS was employed to treat a patient 3 months after a severe traumatic brain injury. This study is particularly notable for its use of advanced imaging techniques to monitor the effects of the intervention, demonstrating significant improvements in the patient's consciousness levels. The successful application of short-term SCS in this case highlights the potential of neuromodulation therapies in enhancing neural activity and promoting recovery in DoC patients. Further exploring this neuromodulation technique, “*Effects of short-term spinal cord stimulation on patients with prolonged disorder of consciousness: A pilot study*” by Zhuang et al. extends the investigation to a larger cohort including 31 patients with DoC (3–23 months post-injury), providing valuable insights into the safety, efficacy, and the specific modulation characteristics of different SCS frequencies. This research emphasizes the importance of tailoring neuromodulation therapies to individual patient needs, potentially leading to more effective and personalized treatment strategies for prolonged DoC. Finally, “*Clinical effect of short-term spinal cord stimulation in the treatment of patients with primary brainstem hemorrhage-induced disorders of consciousness*” by Huang et al. focuses on a specific subset of DoC patients (*n* = 14, 1–1.7-month post-injury), those with primary brainstem hemorrhage-induced conditions. The findings indicate that short-term SCS can lead to significant improvements in this particularly challenging group, suggesting that neuromodulation therapies may offer new hope for patients with brainstem hemorrhage.

Additionally, two neuromodulation protocols have been proposed. The innovative study protocol outlined by Yoon et al. in “*Safety and therapeutic effects of personalized transcranial direct current stimulation based on electrical field simulation for prolonged disorders of consciousness: study protocol for a multi-center, double-blind, randomized controlled trial*” present a novel approach to tDCS treatment, incorporating individual brain lesion characteristics to tailor interventions. This approach aims to enhance the safety and efficacy of tDCS, representing a significant step toward personalized neuromodulation therapies for DoC patients. The second protocol, entitled “*A protocol for a multicenter randomized and personalized controlled trial using rTMS in patients with disorders of consciousness*” by Vitello et al., presents a detailed evaluation plan for 20 Hz rTMS applied to different key brain regions. This work aims to elucidate the most effective stimulation sites and to characterize responder profiles, thereby also contributing to the development of more personalized and effective treatment strategies for DoC.

Finally, addressing the ethical landscape of severe brain injury management, Kreitzer et al. delve into the challenges of prognostication and communication with patients' families. In their brief research report “*Prognostic humility and ethical dilemmas after severe brain injury: Summary, recommendations, and qualitative analysis of Curing Coma Campaign virtual event proceedings*,” the authors call for a multidisciplinary approach to patient care, emphasizing the importance of transparency, empathy, and collaboration in addressing the ethical dilemmas faced by healthcare professionals in this field.

In conclusion, the collection of articles reviewed provides an overview of current advancements in the diagnosis, management and treatment of coma and DoC. The studies collectively address the complex nature of DoC, assess new therapeutic interventions, and emphasize the importance of precise diagnostic techniques and ethical considerations in patient care. As the field evolves, these articles offer a substantive framework that informs ongoing scientific inquiry and clinical practice, aiming to improve the understanding and management of patients with coma and DoC.

## Author contributions

SM: Conceptualization, Writing—original draft, Writing—review & editing. CN: Writing—review & editing. BE: Writing—review & editing. LP: Writing—review & editing. ND: Writing—review & editing. AS: Writing—review & editing. OG: Conceptualization, Writing—original draft, Writing—review & editing.

